# Pentraxins in Complement Activation and Regulation

**DOI:** 10.3389/fimmu.2018.03046

**Published:** 2018-12-19

**Authors:** Ying Jie Ma, Peter Garred

**Affiliations:** The Laboratory of Molecular Medicine, Department of Clinical Immunology, Section 7631, Rigshospitalet, Faculty of Health and Medical Sciences, University of Copenhagen, Copenhagen, Denmark

**Keywords:** pentraxins, PTX3, CRP, SAP, collectin, the ficolins, complement activation, complement regulation

## Abstract

The complement is the first line of immune defense system involved in elimination of invading pathogens and dying host cells. Its activation is mainly triggered by immune complexes or pattern recognition molecules (PRMs) upon recognition against non-self or altered self-cells, such as C1q, collectins, ficolins, and properdin. Recent findings have interestingly shown that the pentraxins (C-reactive protein, CRP; serum-amyloid P component, SAP; long pentraxin 3, PTX3) are involved in complement activation and amplification via communication with complement initiation PRMs, but also complement regulation via recruitment of complement regulators, for instance C4b binding protein (C4BP) and factor H (fH). This review addresses the potential roles of the pentraxins in the complement system during infection and inflammation, and emphasizes the underlining implications of the pentraxins in the context of complement activation and regulation both under physiological and pathological conditions.

## Introduction

**Figure 1:**
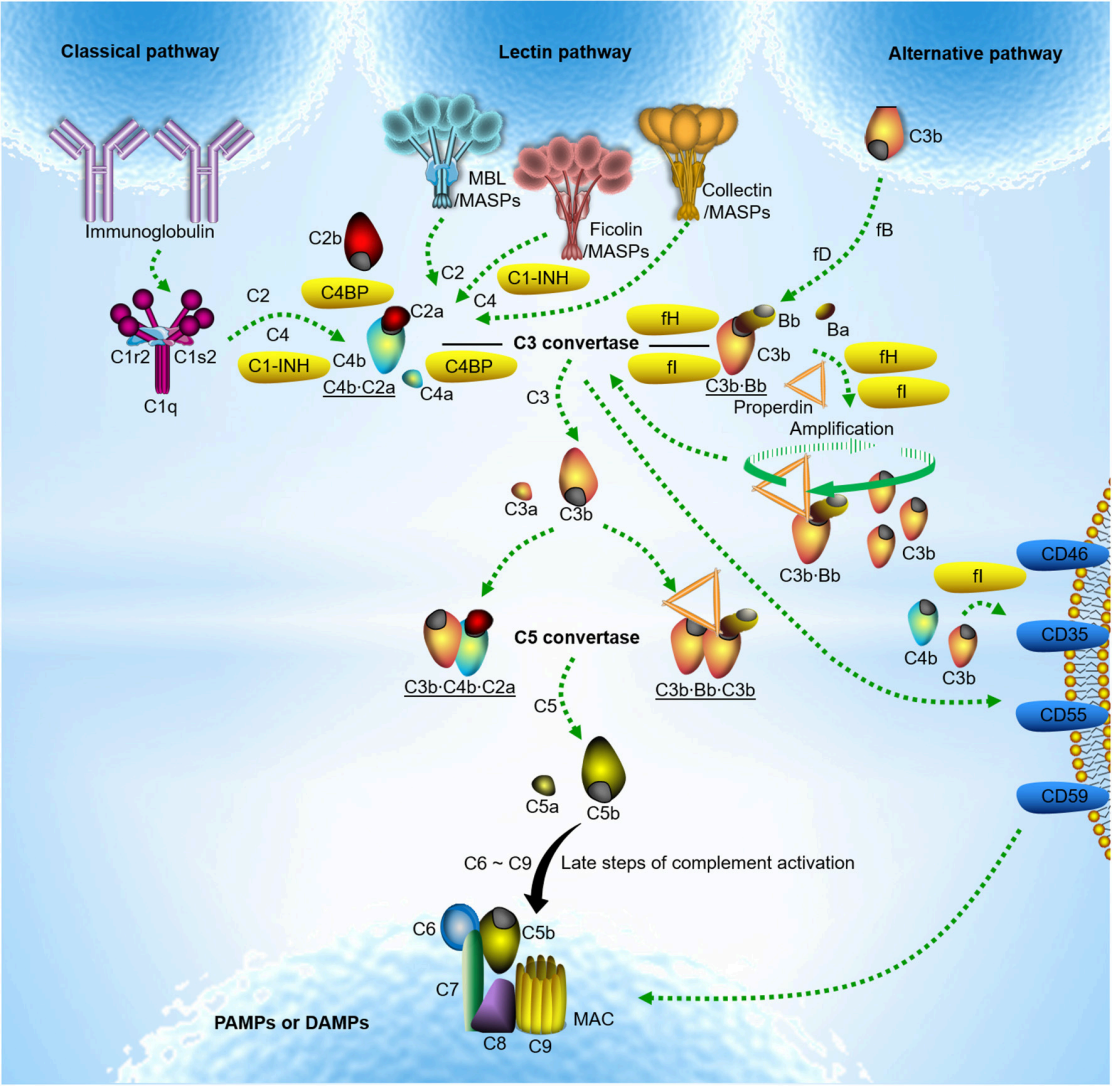
Classical model of complement activation and regulation. Complement activation occurs typically through three routes. Classical pathway activation is triggered by the C1 complex comprising C1q, C1s, and C1r upon binding to IgM or clusters of IgG against pathogen-associated molecular patterns (PAMPs) or damage-associated molecular patterns (DAMPs) (4). Lectin pathway activation is induced by soluble pattern recognition molecules (PRMs) mannose-binding lectin (MBL) upon binding to PAMPs or DAMPs. It is now apparent that the ficolins (ficolin −1,−2, or−3) or collectins (collectin-10, collectin-11 or a heteromeric complex of collectin-10 and collectin-11) are also involved in lectin pathway activation (5–9). Unlike C1q complexed with the serine proteases C1r and C1s, PRMs involving lectin pathway activation are often associated with the mannose-binding lectin-associated serine proteases (MASPs). Upon classical and lectin pathway activation, the serine proteases cleave C4 and C2 to form C3 convertase (C4b·C2a). In contrast, alternative pathway activation occurs by direct tick-over activation of C3 thioester in solution regardless of trigger, and creates its own C3 convertase (C3b·Bb) when activated C3b covalently bind to the target surfaces in contact with factor B (fB) and the enzyme factor D (fD) (10, 11). The alternative C3 convertase is highly stabilized when properdin is associated. With release of anaphylatoxin C3a, surface-bound C3 convertases generate more opsonin C3b, leading to the formation of the classical and lectin pathway C5 convertase (C3b·C4b·C2a) and the alternative C5 convertase (C3b·Bb·C3b). The C5 convertase in turn cleaves C5 into another anaphylatoxin C5a and C5b. Surface-deposited C5b sequentially recruits complement subunits C6, C7, C8, and C9 on target surface and initiates formation of C5b-9 membrane attack complex (MAC) also named the terminal complement complex (TCC) which may lead to target lysis (3, 12). The alternative pathway also serves to amplify classical and lectin pathway activation. Fluid-phase and cell-bound regulators help to modulate complement over-activation; C1 inhibitor (C1-INH) controls the functions of C1r, C1s and MASP-2; C3b and C4b are inactivated by either fluid-phase factor H (fH)/C4b-binding protein (C4BP) or cell-bound complement receptor type 1 (CR1)/CD46 as cofactors for factor I (fI). Fluid-phase fH/C4BP or cell-bound CR1/CD55 regulate convertase activity by disassembly through decay-accelerating activity of the regulators. The formation of MAC is controlled by CD59 (3).

The complement system is one of the ancient innate immune defense system, and can evolutionarily be traced back from the sea urchins (1). In humans, the complement system was initially discovered in 1895 as a heat-labile effector of antibody-mediated immunity. Since then, complement has experienced more than 100 years to unveil its authentic features (2). Today, complement is not only a driver of innate immunity, its functions even extend to additional physiological and/or pathophysiological roles in immune surveillance and homeostasis far beyond simple antimicrobial effector functions (3). Complement exerts its functions through effective rules of activation and regulation under precise control of balance. The part of activation comprises three routes: the classical pathway (CP), the lectin pathway (LP) and the alternative pathway (AP) (Figure 1). However, the complement system has the potential to harm the host if it is not properly controlled and regulated. Therefore, complement activation is precisely modulated in the circulation and on the healthy host cells by exclusive fluid-phase and cell-bound regulators, which are crucial in protecting host cells from complement over-activation (Figure 1).

## Pentraxins: CRP, SAP, and PTX3

Pentraxins are conserved multifunctional soluble pattern recognition molecules (PRMs) characterized by a C-terminal pentraxin signature containing a conserved eight amino acid sequence (13). Proteins of pentraxin family comprise three major members, C-reactive protein (CRP), serum-amyloid P component (SAP), and pentraxin 3 (PTX3). Based on the primary structure of the protomer, CRP and SAP are distinguished as the short pentraxins from the main long pentraxin PTX3 (14). The genes encoding the short pentraxins (CRP/SAP) and the long pentraxin (PTX3) are located on chromosome 1q23 and 3q25, respectively. However, in contrast to the short pentraxins, which are expressed in liver upon stimulation of inflammatory cytokine, the long pentraxin PTX3 is produced by diverse immune cells, such as macrophages, neutrophils, dendritic cells, and endothelial cells (15). CRP is one of the major acute phase proteins in human, and its level in plasma elevates by 1,000-fold via stimulation of hepatocytes by acute phase stimulus. By contrast, PTX3 is barely detectable in human blood circulation (< 2 ng/ml) under physiological conditions, while the serum level drastically increases (200–800 ng/ml) within 6–8 h during infection and inflammation (15). The pentraxins share the conserved C-terminal pentraxin domain, whereas PTX3 harbors a unique N-terminal region without sequence homology to any known proteins so far (15).

The pentraxins recognize wide spectrum of microbial moieties to mediate opsonophargocytosis, but also interact with extracellular matrix (ECM) proteins to stabilize ECM deposition and modified self-structures on dying host cells to keep homeostasis (14). Concerning the antibody-like common features conserved in evolution, the pentraxins mediate phagocytosis and inflammation by macrophages via interaction with the surface Fcγ receptors upon target opsonization (16–18). Thus, the pentraxins are important for both innate immune defense and tissue homeostasis (13, 14, 19).

## Pentraxins-Mediated Complement Cross-activation

Decades of initial research have solidified complement activation by three separate and autonomous routes as described in Figure 1. The CP and LP activation are mainly mediated by exclusive PRMs upon opsonization. Apart from immunoglobulins and several other ligands the CP is also activated via the antibody-like common features of the pentraxins, where CRP, SAP, and PTX3 instead of immunoglobulins recruit C1 complexes upon binding to target surfaces (16, 20, 21). Following antigen stimulation, antibody production often experiences complexity of multiple process by B-cells via differentiation and proliferation and antibody interaction is highly dependent on its antigen specificity. In contrast, the pentraxins are either acute phase reactants under infectious and inflammatory conditions or present constitutively with invariant and high level, and broadly recognize the common pattern moieties arising from microorganisms. Therefore, pentraxins-mediated immune responses are often mobilized rapidly and drastically in resistant to microbial invasion and tissue damage, acting at systemic and local tissue level. Although the antibody-like features have been endowed with the pentraxins in evolution, it is still deficient in antigen-exclusive antibody specificity. Considering complement activation and the underlying functional consequences, pentraxin-mediated initiation is much more rapid and efficient than antibody-dependent responses during infection at early stage (14).

As has been shown for C1q it has recently been shown that the pentraxins interact with some of the PRMs from LP, thus allowing CRP, SAP, and PTX3 to effectively dock at certain bacteria through sensory inputs due to their spontaneous defect in opsonization toward it (22). The interaction has been shown to specifically elevate host immune defense via the LP of complement activation against various microorganism including bacteria and fungi, thus adapting to tricky pathogenic conditions (22). More importantly, the CP and LP, which were previously defined separate and autonomous, have been demonstrated to establish cross-communication through the interaction between the pentraxins and LP PRMs, enabling amplification of complement activation and its concomitant anti-microbial activity (23). In this respect, it should be emphasized that the interaction between the pentraxins and LP PRMs not only serves to boost complement activation, may also result in broadening repertoire of pattern recognition and complement-mediated effector functions via such synergistic effects (24).

**Figure 2:**
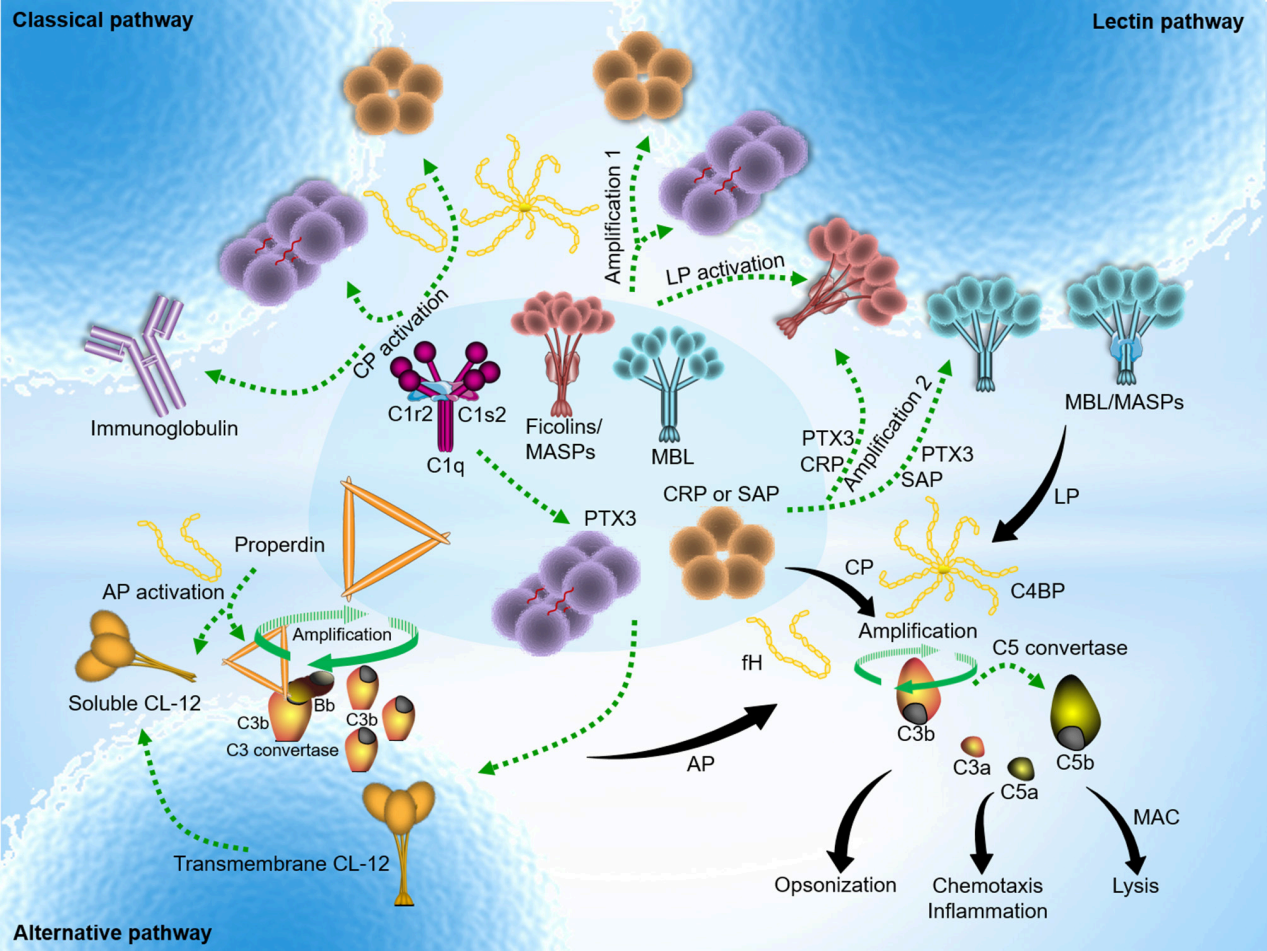
Pentraxins-expanded network of complement activation and regulation. Classical pathway (CP) activation mainly occurs by immune complexes, but also mediated through antibody-like features of the pentraxins in complex with C1q. In addition to the traditional activating fashion as depicted above, lectin pathway (LP) is also indirectly activated by PTX3, CRP, or SAP upon recruitment of MBL or the ficolins. Cross-activation of the CP and LP initiates when heterocomplexes are created on target surfaces between PTX3 (or CRP) and MBL (or ficolin-2) and C1q. Two additional activation pathways emerge to boost complement amplification. The amplification pathways comprise the avenue open to the sequence below: target → PTX3 (or CRP) → ficolin-2/MASPs → C4 → C3 → formation of membrane attach complex (MAC) (amplification 1); target → MBL (or ficolin-2/MASPs) → PTX3 (or CRP) → C1q → C4 → C3 → formation of MAC (amplification 2) (3). Alternative pathway (AP) activation often occurs independently regardless of trigger by spontaneous C3 activation in solution, and rapidly propels nascent C3b binding to nearby target surfaces covalently. In addition, it is hypothesized that surface-bound collectin-12 (CL-12) mediates CP activation by crosstalk with PTX3, CRP or SAP. A recent emerging activation avenue follows relatively specific sequence that involves pattern recognition and opsonization by soluble CL-12 → recruitment of properdin → *de novo* C3 convertase assembly → C3 amplification loop → generation of downstream effector molecules → induction of immune signaling (28). Despite its diverse avenues, overarching concept of complement activation focuses on sensing and eliminating potential danger signals through immunesurveillance and immune effector mechanisms. The pentraxins recognize major fluid-phase complement regulators, C4BP and fH, resulting in down-regulation of complement-mediated inflammation.

The pentraxins are capable of selectively opsonizing certain bacteria, fungi, and viruses (25), but not *Candida albicans* and *Burkholderia cepacia* (26). However, the purified pentraxins (PTX3 and SAP in particular, but not CRP) could bind to *Candida albicans* only in the premise of presence of human serum (27), implying that the presence of certain human serum factor might enable anchorage of the pentraxins on *Candida albicans* indirectly. Recent findings have shown that serum MBL docks both PTX3 and SAP to *Candida albicans*, and that this interaction enhances complement activation and the subsequent opsonophagocytosis by polymorphonuclear leukocytes (PMN) (27). Interaction of PTX3 with MBL leads to communication between the LP and CP via C1q, whereas it is still enigma how SAP:MBL complexes boost complement activation. These findings suggest that crosstalk between the pentraxins and MBL provides two potential complement amplification mechanisms via cross-activation within the complement system (Figure 2).

It has been well documented that the complement system plays a non-redundant role against *Aspergillus fumigatus* infection (29). The state of complement deficiency is highly susceptible to *Aspergillus fumigatus* infection and mice deficient in C5 has been shown to be hardly survived (30, 31). Ficolin-2 has been recently suggested to serve as a particular inducer of anti-fungal activity through provoking the LP of complement activation and/or regulating pro- and anti-inflammatory airway immune responses in treatment of *Aspergillus fumigatus* challenge (24, 32, 33). Ficolin-2 has been shown to recognize *Aspergillus fumigatus* in a Ca^2+^-insensitive manner with stronger binding at acidic pH (34, 35), which typically prevails in a local infectious and inflammatory condition (36) and is required to boost complement activity (37). Zhang et al. previously reported that interaction of CRP with ficolin-2 is elevated in an acidic pH-sensitive manner (23). These data suggest that local prevalence of acidic circumstances may be essential to trigger reciprocal interaction between ficolin-2 and CRP to combat *Aspergillus fumigatus* at the early stage of infection. Consistent with the previous reports, analysis of bronchoalveolar lavage (BAL) fluid has attested presence of ficolin-2 in invasive aspergillosis (IA)-suffering patients, and demonstrated a notable roles of ficolin-2 in modulation of alveolar immune responses against infection of *Aspergillus fumigatus* (35). In agreement with those reports, Genster et al. recently found that mice are vulnerable to fungal infection under the ficolin-deficient condition (38). These results suggest that serum ficolin-2 may facilitate to elevate host immune responses at local sites of pulmonary fungal infection via transmigration to alveoli and thus play a crucial role in lung infection (35). Mice deficient in PTX3 have shown increased susceptibility to invasive pulmonary aspergillosis (IPA) and accelerated death compared with mock control, which was attributed to the defect in fungicidal activities against IPA in regard to opsonophagocytosis and activation of an adaptive type 2 responses (39). In parallel with those evidences, Cunha et al. intriguingly found a PTX3 single-nucleotide polymorphisms (SNPs) in donors with a homozygous haplotype, leading to increased vulnerability to invasive aspergillosis when patients receives hematopoietic stem-cell transplantation from such donors (39). These results suggest that PTX3 plays non-redundant roles in antifungal immunity. In this respect, it should be noted that collaboration of liver-derived CRP and immune cell-expressed PTX3 with migratory ficolin-2 might also further boost the microbicidal immune responses at systemic and local tissue level, respectively, for instance through the complement cross-activation and amplification mechanisms.

Recently, the pentraxins have been shown to interact with CL-12, a newly identified scavenger receptor C-type lectin (SRCL) (40, 41). By using ELISA and CHO/ldlA7 cell lines expressing transmembrane CL-12 as platform, the interaction of the pentraxins with transmembrane CL-12 has been visualized. It was shown a propensity of the pentraxins including CRP, SAP, and PTX3 for direct binding of CL-12, and demonstrated that the interaction is able to result in the CP of complement activation via recruitment of C1q on HEK293 cell lines expressing transmembrane CL-12 (42). However, whether this is indeed a case on native CL-12-expressing primary cells, for instance HUVEC or HUAEC, is still awaiting further clarification. Nevertheless, a soluble form of CL-12 has recently been identified and revealed to trigger AP of complement activation through direct contact with properdin (28). Whether the pentraxins interact with soluble form of CL-12 and expand a novel complement crosstalk against invading pathogens is still unknown.

## Pentraxins in Complement Regulation

In general, complement activation is precisely modulated in the circulation and on healthy host cells by exclusive soluble and cell-bound regulators as described in Figure 1. Recent data show that the pentraxins surveil and modulate the action of complement to avoid overwhelming activation via interaction with complement negative regulators. Like CRP and SAP, PTX3 is able to bind the main fluid-phase regulator of the CP and LP C4BP. Similar to the short pentraxins, PTX3 in solution preserve the cofactor activity of C4BP for fI upon complex formation (43). When anchored on apoptotic/necrotic cells and extracellular matrix (ECM), PTX3 is capable of recruiting functionally active C4BP, leading to complement C4b inactivation and reduced terminal complement complex (TCC) deposition on the surfaces. Both CRP and PTX3 have been observed to interact with fH, the main soluble regulator of the AP, and the two PTX3 binding sites on fH were defined to be located on fH short consensus repeat (SCR) 7 and SCR19-20 (44), of which the former is also employed for CRP binding in addition to SCR8-11 (45, 46). fH was found to remain its C3 inhibitory activity upon binding to the pentraxins, thus preventing exaggerated AP mediated complement activation on CRP or PTX3-immobilized surfaces. Therefore, the pentraxins may target all the complement pathways by interaction with C4BP and fH and they may assist in regulation of complement activation to avoid the adverse effect of complement in tissues.

Mutations or polymorphisms of fH are associated with the pathogenesis of various inflammatory human diseases, for instance atypical hemolytic uremic syndrome (aHUS) and age-related macular degeneration (AMD) due to dysregulation of the AP. Interestingly, Tyr402His (a polymorphic amino acid variant in SCR7 of fH), which is linked to high risk of aHUS and AMD, influences the binding of CRP, but not PTX3-fH interaction. Therefore, reduced CRP- fH Tyr402His interaction might be involved in pathogenesis of the diseases due to the complement-mediated increased inflammation, where PTX3 could compensate for these changes at the site of inflammation. Kelly et al. previously showed that heparin sulfate inhibits the AP through interaction with fH, and found that fH-mediated C3b inactivation is highly dependent on heparin sulfate and the degree of sulphation in the Bruch's membrane (BrM) (47). Thus, BrM/choroid, a site of tissue damage expressing abundant heparin sulfate glycosaminoglycans in AMD, might be protected by recruitment of fH under normal conditions. In addition, PTX3 stored inside BrM might also act as a second line protection for mobilization of fH and modulate complement activation. Given the following facts that (1) the binding site for heparin on fH is overlapped with PTX3 to SCR19-20; (2) fH Tyr402His influences the binding of heparin as compared with its wild type; (3) PTX3 binds equally to both the variant and the wild type, the state of PTX3 deficiency would impact on the patients more with fH Tyr402His than its wild type, since the decline in fH binding to heparin would not be supplied by reserved PTX3. In this respect, it is worthy to note that since PTX3 is rapidly and dramatically expressed as an acute phase reactant in the retinal pigmented epithelium (RPE) in response to inflammatory stimuli, the manifestations of fH Tyr402His might be veiled in AMD. Interestingly, it has been recently substantiated that PTX3 serves to brake the complement and the subsequent NLRP3 inflammasome activation through regulation of fH in the RPE (48).

Malignant cells have been previously shown to be monitored and recognized by the complement system and the concomitant complement activation often occurs in many cancers upon recognition (49). However, the complement system has also been suggested to have a role in the development of tumor promoting inflammation and the intermediate product of complement activation C5a has been shown to play a tumor-exacerbating role via elevation of T cell suppression effect and CCL-2 production attracting tumor-associated macrophages (TAMs) and favoring M2-like polarization by bone marrow-derived suppressor cells (50, 51). PTX3 has recently been shown to contribute in regulation of tumor growth, which was attributed to its capacity in control of tumor promoting inflammation through coordination with complement regulator fH (52). As such PTX3 does seem to have a direct effect on tumor cell growth. However, PTX3 deficient animals were more susceptible to chemically induced mesenchymal and epithelial carcinogenesis than control animals (52). PTX3 deficient tumors showed enhanced complement C3 deposition and C5a levels, CCL2 production, and tumor-promoting macrophage recruitment, which was attributed to dysregulated complement activation since C3-genetic inactivation and CCL2 inhibition reverted the phenotype and the increased susceptibility to mesenchymal carcinogenesis in PTX3-deficient mice. These findings suggest a crucial role of PTX3 in complement-dependent tumor-related inflammation probably via fH recruitment. Lack of the functional PTX3 and/or fH might exacerbate pathophysiological consequences during tumor growth and development due to complement dysregulation.

Apart from being a serum marker of systemic inflammation CRP is also suggested to have a direct pathological role in tissues in diseases, such as AMD (53, 54). CRP exists primarily in two forms with distinct structure and biological activity, the native pentameric CRP (pCRP) and monomeric CRP (mCRP), and circulates in the blood stream as a non-covalent, anti-inflammatory pCRP, however when dissociated into mCRP upon binding to certain cell and matrix surfaces, it becomes rather pro-inflammatory (55). Given the findings that the majority of CRP found in BrM is in monomeric form, although pCRP will still be present in the blood vessels of the choroid *in vivo* (53), mCRP is likely a tissue-associated form derived from circulating pCRP. Accumulating evidences show that if fH is dysfunctional mCRP activates the complement at retina/choroid interface and leads to exacerbate chronic inflammation and subsequent tissue damage (56), indicating the pivotal role of pro-inflammatory mCRP-mediated complement activation and regulation in normal conditions. In addition, fH has been shown to interact with mCRP through its binding sites located in SCR 6-8 and SCR 19-20 regions, but not with pCRP (46, 57–61).

## Concluding Remarks

The complement system is a double-edged sword, one toward to eliminate danger signals and hostile intruders, while the other is toward healthy self, which may lead to pathological situations if it is not controlled properly. The pentraxins are involved in both complement activation and regulation via crosstalk with major complement initiating molecules (C1q, ficolin-2, MBL, and CL-12) as well as complement regulators (C4BP and fH) (Figure 2). The crosstalk events between the pentraxins and the CP and LP PRMs have been recently highlighted for their amplifying roles in immunosurveillance, anti-microbial immune responses, and immunologic homeostasis. However, evidence suggests immunopathogenicity of those cooperative events in infectious inflammation if activated inappropriately, and also emphasizes the potential deleterious impact on pathogen immune evasion and development of complement-related diseases. The pathogenic side of the potential functional roles of PRM heterocomplexes and their involvement in complement activation need to be further explored: whether PRM heterocomplexes are involved in potent inducers of immunopathology during infection and inflammation and how it exacerbates disease severity are both intriguing unanswered questions. Furthermore, recent work has focused on how the pentraxins-mediated complement activation and regulation influences and thus contributes to chronic inflammation, which likely constructs a microenvironment for initiation and development of complement-mediated pathology. Emerging evidences discussed above reinforce the importance of the pentraxins (CRP and PTX3 in particular) in complement activation and regulation in the pathogenesis of age-associated diseases and complement-dependent tumor-promoting inflammation. Given the recent findings that novel collectin 11 or properdin-directed complement activation triggers acute kidney injury, it is also interesting to determine whether and how the pentraxins regulate or exacerbate complement-involved renal injury upon complex formation with the inducers of renal injury. After many years' success of complement drug applications in the treatment of complement-related diseases (ex. PNH and aHUS), the future of the complement field becomes much brighter and many of complement drugs are being processed in preclinical and clinical stages of development in current years. Pentraxins are recently positioning with renewed focus as a novel therapeutic targets being explored in the community of drug development. Importantly, anti-pentraxins drugs (CPHPC and dezamizumab) targeting human SAP have recently entered in clinical phase 2 trial for treatment of amyloidosis and Alzheimer's disease, and human CRP inhibitor targeting CRP-driven complement activation-mediated tissue damage is also being developed (62). Taken together, a better understanding of the complex roles of the pentraxins in the complement system and its involvement in human inflammatory diseases will direct more promising options of therapy against the consequences of certain pathogen infection, and possibly certain complement-related inflammatory diseases.

## Author Contributions

YM and PG conceived, designed and wrote the review article. YM and PG revised the review article and approved the final manuscript.

### Conflict of Interest Statement

The authors declare that the research was conducted in the absence of any commercial or financial relationships that could be construed as a potential conflict of interest.

## References

[1] Nonaka M Yoshizaki F. Evolution of the complement system. Mol Immunol. (2004) 40:897–902. 10.1007/978-94-017-8881-6_3 14698228

[2] Walport MJ. Complement. First of two parts. N Engl J Med. (2001) 344:1058–66. 10.1056/NEJM200104053441406 11287977

[3] Ricklin D Hajishengallis G Yang K Lambris JD. Complement: a key system for immune surveillance and homeostasis. Nat Immunol. (2010) 11:785–97. 10.1038/ni.1923 20720586 PMC2924908

[4] Gaboriaud C Thielens NM Gregory LA Rossi V Fontecilla-Camps JC Arlaud GJ. Structure and activation of the C1 complex of complement: unraveling the puzzle. Trends Immunol. (2004) 25:368–73. 10.1016/j.it.2004.04.008 15207504

[5] Garred P Honore C Ma YJ Munthe-Fog L Hummelshoj T. MBL2, FCN1, FCN2 and FCN3-The genes behind the initiation of the lectin pathway of complement. Mol Immunol. (2009) 46:2737–44. 10.1016/j.molimm.2009.05.005 19501910

[6] Henriksen ML Brandt J Andrieu JP Nielsen C Jensen PH Holmskov U. Heteromeric complexes of native collectin kidney 1 and collectin liver 1 are found in the circulation with MASPs and activate the complement system. J Immunol. (2013) 191:6117–27. 10.4049/jimmunol.1302121 24174618

[7] Axelgaard E Jensen L Dyrlund TF Nielsen HJ Enghild JJ Thiel S. Investigations on collectin liver 1. J Biol Chem. (2013) 288:23407–20. 10.1074/jbc.M113.492603 23814060 PMC3743509

[8] Ma YJ Skjoedt MO Garred P. Collectin-11/MASP complex formation triggers activation of the lectin complement pathway–the fifth lectin pathway initiation complex. J Innate Immun. (2013) 5:242–50. 10.1159/000345356 23220946 PMC6741501

[9] Garred P Genster N Pilely K Bayarri-Olmos R Rosbjerg A Ma YJ. A journey through the lectin pathway of complement-MBL and beyond. Immunol Rev. (2016) 274:74–97. 10.1111/imr.12468 27782323

[10] Alcorlo M Tortajada A Rodriguez De Cordoba S Llorca O. Structural basis for the stabilization of the complement alternative pathway C3 convertase by properdin. Proc Natl Acad Sci USA. (2013) 110:13504–9. 10.1073/pnas.1309618110 23901101 PMC3746899

[11] Fearon DT Austen KF. Properdin: binding to C3b and stabilization of the C3b-dependent C3 convertase. J Exp Med. (1975) 142:856–63. 1185108 10.1084/jem.142.4.856 PMC2189935

[12] Ma YJ Lee BL Garred P. An overview of the synergy and crosstalk between pentraxins and collectins/ficolins: their functional relevance in complement activation. Exp Mol Med. (2017) 49:e320. 10.1038/emm.2017.51 28428631 PMC6130212

[13] Garlanda C Bottazzi B Bastone A Mantovani A. Pentraxins at the crossroads between innate immunity, inflammation, matrix deposition, and female fertility. Annu Rev Immunol. (2005) 23:337–66. 10.1146/annurev.immunol.23.021704.115756 15771574

[14] Bottazzi B Doni A Garlanda C Mantovani A. An integrated view of humoral innate immunity: pentraxins as a paradigm. Annu Rev Immunol. (2010) 28:157–83. 10.1146/annurev-immunol-030409–101305 19968561

[15] Mantovani A Garlanda C Doni A Bottazzi B. Pentraxins in innate immunity: from C-reactive protein to the long pentraxin PTX3. J Clin Immunol. (2008) 28:1–13. 10.1007/s10875-007-9126-7 17828584

[16] Bottazzi B Vouret-Craviari V Bastone A De Gioia L Matteucci C Peri G. Multimer formation and ligand recognition by the long pentraxin PTX3. Similarities and differences with the short pentraxins C-reactive protein and serum amyloid P component. J Biol Chem. (1997) 272:32817–23. 9407058 10.1074/jbc.272.52.32817

[17] Nauta AJ Bottazzi B Mantovani A Salvatori G Kishore U Schwaeble WJ. Biochemical and functional characterization of the interaction between pentraxin 3 and C1q. Eur J Immunol. (2003) 33:465–73. 10.1002/immu.200310022 12645945

[18] Baruah P Dumitriu IE Peri G Russo V Mantovani A Manfredi AA. The tissue pentraxin PTX3 limits C1q-mediated complement activation and phagocytosis of apoptotic cells by dendritic cells. J Leukoc Biol. (2006) 80:87–95. 10.1189/jlb.0805445 16617159

[19] Garlanda C Bottazzi B Magrini E Inforzato A Mantovani A. PTX3, a Humoral Pattern Recognition Molecule, in Innate Immunity, Tissue Repair, and Cancer. Physiol Rev. (2018) 98:623–39. 10.1152/physrev.00016.2017 29412047 PMC5985957

[20] Nauta AJ Daha MR Van Kooten C Roos A. Recognition and clearance of apoptotic cells: a role for complement and pentraxins. Trends Immunol. (2003) 24:148–54. 10.1016/S1471-4906(03)00030-9 12615211

[21] Gershov D Kim S Brot N Elkon KB. C-Reactive protein binds to apoptotic cells, protects the cells from assembly of the terminal complement components, and sustains an antiinflammatory innate immune response: implications for systemic autoimmunity. J Exp Med. (2000) 192:1353–64. 10.1084/jem.192.9.1353 11067883 PMC2193350

[22] Ng PM Le Saux A Lee CM Tan NS Lu J Thiel S. C-reactive protein collaborates with plasma lectins to boost immune response against bacteria. EMBO J. (2007) 26:3431–40. 10.1038/sj.emboj.7601762 17581635 PMC1933394

[23] Zhang J Koh J Lu J Thiel S Leong BS Sethi S. Local inflammation induces complement crosstalk which amplifies the antimicrobial response. PLoS Pathog. (2009) 5:e1000282. 10.1371/journal.ppat.1000282 19180241 PMC2629585

[24] Ma YJ Doni A Hummelshoj T Honore C Bastone A Mantovani A. Synergy between ficolin-2 and pentraxin 3 boosts innate immune recognition and complement deposition. J Biol Chem. (2009) 284:28263–75. 10.1074/jbc.M109.009225 19632990 PMC2788878

[25] Deban L Jaillon S Garlanda C Bottazzi B Mantovani A. Pentraxins in innate immunity: lessons from PTX3. Cell Tissue Res. (2011) 343:237–49. 10.1007/s00441–010-1018–0 20683616

[26] Garlanda C Hirsch E Bozza S Salustri A De Acetis M Nota R. Non-redundant role of the long pentraxin PTX3 in anti-fungal innate immune response. Nature (2002) 420:182–6. 10.1038/nature01195 12432394

[27] Ma YJ Doni A Skjoedt MO Honore C Arendrup M Mantovani A. Heterocomplexes of mannose-binding lectin and the pentraxins PTX3 or serum amyloid P component trigger cross-activation of the complement system. J Biol Chem. (2011) 286:3405–17. 10.1074/jbc.M110.190637 21106539 PMC3030347

[28] Ma YJ Hein E Munthe-Fog L Skjoedt MO Bayarri-Olmos R Romani L. Soluble collectin-12 (CL-12) is a pattern recognition molecule initiating complement activation via the alternative pathway. J Immunol. (2015) 195:3365–73. 10.4049/jimmunol.1500493 26290605

[29] Hector RF Yee E Collins MS. Use of DBA/2N mice in models of systemic candidiasis and pulmonary and systemic aspergillosis. Infect Immun. (1990) 58:1476–8. 2323826 10.1128/iai.58.5.1476-1478.1990 PMC258651

[30] Svirshchevskaya EV Shevchenko MA Huet D Femenia F Latge JP Boireau P. Susceptibility of mice to invasive aspergillosis correlates with delayed cell influx into the lungs. Int J Immunogenet. (2009) 36:289–99. 10.1111/j.1744-313X.2009.00869.x 19744035

[31] Henwick S Hetherington SV Patrick CC. Complement binding to Aspergillus conidia correlates with pathogenicity. J Lab Clin Med. (1993) 122:27–35. 8320488

[32] Bidula S Kenawy H Ali YM Sexton D Schwaeble WJ Schelenz S. Role of ficolin-A and lectin complement pathway in the innate defense against pathogenic Aspergillus species. Infect Immun. (2013) 81:1730–40. 10.1128/IAI.00032-13 23478320 PMC3647983

[33] Bidula S Sexton DW Schelenz S. Serum opsonin ficolin-A enhances host-fungal interactions and modulates cytokine expression from human monocyte-derived macrophages and neutrophils following Aspergillus fumigatus challenge. Med Microbiol Immunol. (2016) 205:133–42. 10.1007/s00430-015-0435-9 26337048

[34] Schelenz S Kirchhof N Bidula S Wallis R Sexton DW. Opsonizing properties of rat ficolin-A in the defence against Cryptococcus neoformans. Immunobiology (2013) 218:477–83. 10.1016/j.imbio.2012.06.006 22789560

[35] Bidula S Sexton DW Abdolrasouli A Shah A Reed A Armstrong-James D. The serum opsonin L-ficolin is detected in lungs of human transplant recipients following fungal infections and modulates inflammation and killing of Aspergillus fumigatus. J Infect Dis. (2015) 212:234–46. 10.1093/infdis/jiv027 25612732

[36] Martínez D Vermeulen M Trevani A Ceballos A Sabatté J Gamberale R. Extracellular acidosis induces neutrophil activation by a mechanism dependent on activation of phosphatidylinositol 3-kinase/Akt and ERK pathways. J Immunol. (2006) 176:1163–71. 10.4049/jimmunol.176.2.1163 16394005

[37] Kenawy HI Boral I Bevington A. Complement-coagulation cross-talk: a potential mediator of the physiological activation of complement by low pH. Front Immunol. (2015) 6:215. 10.3389/fimmu.2015.00215 25999953 PMC4422095

[38] Genster N Praestekjaer Cramer E Rosbjerg A Pilely K Cowland JB Garred P. Ficolins promote fungal clearance *in vivo* and modulate the inflammatory cytokine response in host defense against Aspergillus fumigatus. J Innate Immun. (2016) 8:579–88. 10.1159/000447714 27467404 PMC6738752

[39] Cunha C Aversa F Lacerda JF Busca A Kurzai O Grube M. Genetic PTX3 deficiency and aspergillosis in stem-cell transplantation. N Engl J Med. (2014) 370:421–32. 10.1056/NEJMoa1211161 24476432

[40] Nakamura K Funakoshi H Miyamoto K Tokunaga F Nakamura T. Molecular cloning and functional characterization of a human scavenger receptor with C-type lectin (SRCL), a novel member of a scavenger receptor family. Biochem Biophys Res C (2001) 280:1028–35. 10.1006/bbrc.2000.4210 11162630

[41] Ohtani K Suzuki Y Eda S Kawai T Kase T Keshi H. The membrane-type collectin CL-P1 is a scavenger receptor on vascular endothelial cells. J Biol Chem. (2001) 276:44222–8. 10.1074/jbc.M103942200M103942200[pii] 11564734

[42] Roy N Ohtani K Hidaka Y Amano Y Matsuda Y Mori K. Three pentraxins C-reactive protein, serum amyloid p component and pentraxin 3 mediate complement activation using Collectin CL-P1. Biochim Biophys Acta (2017) 1861:1–14. 10.1016/j.bbagen.2016.11.023 27864148

[43] Braunschweig A Jozsi M. Human pentraxin 3 binds to the complement regulator c4b-binding protein. PLoS ONE (2011) 6:e23991. 10.1371/journal.pone.0023991 21915248 PMC3161823

[44] Deban L Jarva H Lehtinen MJ Bottazzi B Bastone A Doni A. Binding of the long pentraxin PTX3 to factor H: interacting domains and function in the regulation of complement activation. J Immunol. (2008) 181:8433–40. 10.4049/jimmunol.181.12.8433 19050261

[45] Jarva H Jokiranta TS Hellwage J Zipfel PF Meri S. Regulation of complement activation by C-reactive protein: targeting the complement inhibitory activity of factor H by an interaction with short consensus repeat domains 7 and 8–11. J Immunol. (1999) 163:3957–62. 10490997

[46] Okemefuna AI Nan R Miller A Gor J Perkins SJ. Complement factor H binds at two independent sites to C-reactive protein in acute phase concentrations. J Biol Chem. (2010) 285:1053–65. 10.1074/jbc.M109.044529 19850925 PMC2801232

[47] Kelly U Yu L Kumar P Ding JD Jiang H Hageman GS. Heparan sulfate, including that in Bruch's membrane, inhibits the complement alternative pathway: implications for age-related macular degeneration. J Immunol. (2010) 185:5486–94. 10.4049/jimmunol.0903596 20876352 PMC3639479

[48] Wang L Cano M Datta S Wei H Ebrahimi KB Gorashi Y. Pentraxin 3 recruits complement factor H to protect against oxidative stress-induced complement and inflammasome overactivation. J Pathol. (2016) 240:495–506. 10.1002/path.4811 27659908

[49] Reis ES Mastellos DC Ricklin D Mantovani A Lambris JD. Complement in cancer: untangling an intricate relationship. Nat Rev Immunol. (2018) 18:5–18. 10.1038/nri.2017.97 28920587 PMC5816344

[50] Mantovani A Allavena P Sica A Balkwill F. Cancer-related inflammation. Nature (2008) 454:436–44. 10.1038/nature07205 18650914

[51] Markiewski MM Deangelis RA Benencia F Ricklin-Lichtsteiner SK Koutoulaki A Gerard C. Modulation of the antitumor immune response by complement. Nat Immunol. (2008) 9:1225–35. 10.1038/ni.1655 18820683 PMC2678913

[52] Bonavita E Gentile S Rubino M Maina V Papait R Kunderfranco P. PTX3 is an extrinsic oncosuppressor regulating complement-dependent inflammation in cancer. Cell (2015) 160:700–14. 10.1016/j.cell.2015.01.004 25679762

[53] Chirco KR Whitmore SS Wang K Potempa LA Halder JA Stone EM. Monomeric C-reactive protein and inflammation in age-related macular degeneration. J Pathol. (2016) 240:173–83. 10.1002/path.4766 27376713 PMC5527328

[54] Bhutto IA Baba T Merges C Juriasinghani V Mcleod DS Lutty GA. C-reactive protein and complement factor H in aged human eyes and eyes with age-related macular degeneration. Br J Ophthalmol. (2011) 95:1323–30. 10.1136/bjo.2010.199216 21633121 PMC4916773

[55] Wu Y Potempa LA El Kebir D Filep JG. C-reactive protein and inflammation: conformational changes affect function. Biol Chem. (2015) 396:1181–97. 10.1515/hsz-2015–0149 26040008

[56] Johnson PT Betts KE Radeke MJ Hageman GS Anderson DH Johnson LV. Individuals homozygous for the age-related macular degeneration risk-conferring variant of complement factor H have elevated levels of CRP in the choroid. Proc Natl Acad Sci USA. (2006) 103:17456–61. 10.1073/pnas.0606234103 17079491 PMC1859950

[57] Sjoberg AP Trouw LA Clark SJ Sjolander J Heinegard D Sim RB. The factor H variant associated with age-related macular degeneration (His-384) and the non-disease-associated form bind differentially to C-reactive protein, fibromodulin, DNA, and necrotic cells. J Biol Chem. (2007) 282:10894–900. 10.1074/jbc.M610256200 17293598

[58] Laine M Jarva H Seitsonen S Haapasalo K Lehtinen MJ Lindeman N. Y402H polymorphism of complement factor H affects binding affinity to C-reactive protein. J Immunol. (2007) 178:3831–6. 10.4049/jimmunol.178.6.3831 17339482 PMC4853917

[59] Giannakis E Jokiranta TS Male DA Ranganathan S Ormsby RJ Fischetti VA. A common site within factor H SCR 7 responsible for binding heparin, C-reactive protein and streptococcal M protein. Eur J Immunol. (2003) 33:962–9. 10.1002/eji.200323541 12672062

[60] Mihlan M Stippa S Jozsi M Zipfel PF. Monomeric CRP contributes to complement control in fluid phase and on cellular surfaces and increases phagocytosis by recruiting factor H. Cell Death Differ. (2009) 16:1630–40. 10.1038/cdd.2009.103 19680263

[61] Hakobyan S Harris CL Van Den Berg CW Fernandez-Alonso MC De Jorge EG De Cordoba SR. Complement factor H binds to denatured rather than to native pentameric C-reactive protein. J Biol Chem. (2008) 283:30451–60. 10.1074/jbc.M803648200 18786923 PMC2662140

[62] Pepys MB. The pentraxins 1975–2018: serendipity, diagnostics and drugs. Front Immunol. (2018) 9:2382. 10.3389/fimmu.2018.02382 30459761 PMC6232782

